# Effect of Temperature on Crossbridge Force Changes during Fatigue and Recovery in Intact Mouse Muscle Fibers

**DOI:** 10.1371/journal.pone.0078918

**Published:** 2013-10-17

**Authors:** Marta Nocella, Giovanni Cecchi, Maria Angela Bagni, Barbara Colombini

**Affiliations:** Department of Experimental and Clinical Medicine, University of Florence, Florence, Italy; University of Rome La Sapienza, Italy

## Abstract

Repetitive or prolonged muscle contractions induce muscular fatigue, defined as the inability of the muscle to maintain the initial tension or power output. In the present experiments, made on intact fiber bundles from FDB mouse, fatigue and recovery from fatigue were investigated at 24°C and 35°C. Force and stiffness were measured during *tetani* elicited every 90 s during the pre-fatigue control phase and recovery and every 1.5 s during the fatiguing phase made of 105 consecutive *tetani*. The results showed that force decline could be split in an initial phase followed by a later one. Loss of force during the first phase was smaller and slower at 35°C than at 24°C, whereas force decline during the later phase was greater at 35°C so that total force depression at the end of fatigue was the same at both temperatures. The initial force decline occurred without great reduction of fiber stiffness and was attributed to a decrease of the average force per attached crossbridge. Force decline during the later phase was accompanied by a proportional stiffness decrease and was attributed to a decrease of the number of attached crossbridge. Similarly to fatigue, at both 24 and 35°C, force recovery occurred in two phases: the first associated with the recovery of the average force per attached crossbridge and the second due to the recovery of the pre-fatigue attached crossbridge number. These changes, symmetrical to those occurring during fatigue, are consistent with the idea that, i) initial phase is due to the direct fast inhibitory effect of [P_i_]_i_ increase during fatigue on crossbridge force; ii) the second phase is due to the delayed reduction of Ca^2+^ release and /or reduction of the Ca^2+^ sensitivity of the myofibrils due to high [P_i_]_i_.

## Introduction

Repetitive or prolonged muscle contractions induce muscular fatigue, defined as the inability of the muscle to maintain the initial tension or power output. Prolonged muscle contractions also lead to an increased muscle metabolism and increase muscle temperature *in vivo* well above the resting value. In contrast, studies on skeletal muscle fatigue *in vitro* are often performed at room temperature around 20-24°C, about 10-15°C below the physiological temperature range. Thus it is possible that fatigue characteristics measured in these studies are different from those occurring at physiological temperature. Data in literature show, for example, that the depressant effects of acidosis and intracellular [P_i_] on force generation, both increasing during fatigue, are greater at lower than at higher near-physiological temperatures [1-3]. 

Tetanic force decline of fast fibers during fatigue caused by repetitive stimulation follows a characteristic pattern composed by three phases: an initial one during which force drops and tetanic [Ca^2+^]_i_ slightly increases, a second phase in which tension stays constant or declines slowly and by a third phase during which tension and [Ca^2+^]_i_ decline rapidly [4]. Recently [5] we measured both force and stiffness of mouse flexor digitorum brevis (FDB) fibers during fatigue at 24°C and we found that time course of fatigue could be satisfactorily described by two phases: an initial one (phase 1) during which force dropped quickly by ~20 %, followed by a later phase (phase 2) during which force further decreased to a final ~50% of control. In contrast to force, stiffness decreased little during phase 1 whereas it decreased in parallel with force during phase 2. Consistently with previous suggestions in literature [4], we showed that the initial phase of fatigue was mainly due to reduction of the individual crossbridge force whereas the second phase was better explained by a reduction of attached crossbridge number [5]. Both these effects were attributed to phosphate accumulation inside the fibers caused by creatinphosphate breakdown during repetitive muscular activity. Phosphate would act directly on crossbridges reducing the individual crossbridge force and indirectly through a reduction of calcium release and/or myofibrillar calcium sensitivity which would reduce the number of attached crossbridges [5,6]. Our experiments were carried out at temperature of 24°C, however many of the mechanisms that contribute to fatigue are temperature sensitive and fatigue may be more prominent and occurring more rapidly at high compared to low temperatures. For these reasons we decided to investigate whether the crossbridge mechanisms of fatigue shown at 24°C were also valid at 35°C, close to the *in vivo* temperature of mouse FDB muscle during an intense and prolonged exercise [7-9]. In addition we investigated the mechanism of force recovery from fatigue at both temperatures. The results showed that tension decline during the initial phase of fatigue was smaller and slower at 35°C compared to 24°C, whereas force decline during phase 2 was greater at higher temperature so that force depression after 105 *tetani* was ~50% at both temperatures. Consistently with our previous finding, initial force decline was accompanied by a smaller stiffness decline at both temperatures, whereas stiffness decreased in parallel with tension during the second phase. Similarly, force recovery from fatigue occurred in two phases. Phase one was very fast and it was associated with the recovery of the individual crossbridge force whereas phase two was mainly due to the recovery of the pre-fatigue attached crossbridge number. These changes fully reversed the effects of fatigue.

## Materials and Methods

### Ethic statement

This study was carried out following the EEC guidelines for animal care of The European Community Council (Directive 86/609/EEC) and the protocol was approved by the Ethical Committee for Animal Experiments of the University of Florence (acceptance signed by the veterinary responsible in October 20, 2010). Mice were housed at controlled temperature (21-24°C) with a 12-12 h light-dark cycle. Food and water were provided *ad libitum*. Animals (C57BL/6, male, 3-6 month-old) were killed by rapid cervical dislocation. The number of animals used (n = 7) was minimized by using of more than one preparation from the flexor digitorum brevis (FDB) muscle taken from both legs.

### Fiber preparation and measures

Small bundles of 6-15 fibers were mechanically dissected from the FDB muscle as described previously [10]. The use of small bundles avoids anoxia problems which are possible with the whole muscle and offers good mechanical conditions for fast stiffness measurements. Fibers from this muscle are mainly of IIa and IIx types [11,12]. Small aluminum clips were attached to tendons as close as possible to the fiber ends and were used to mount the fibers horizontally in an experimental chamber (capacity 0.38 ml) between the lever arms of an home-made capacitance force transducer (resonance frequency, 16-30 kHz) and of an electromagnetic motor used to apply fast length changes to the fibers. Fibers were superfused continuously by means of a peristaltic pump at a rate of about 0.35 ml min^−1^ with a Tyrode solution of the following composition (mM): NaCl, 121; KCl, 5; CaCl_2_, 1.8; MgCl_2_, 0.5; NaH_2_PO_4_, 0.4; NaHCO_3_, 24; glucose, 5.5; EDTA, 0.1 and bubbled with 5% CO_2_ - 95% O_2_ which gave a pH of 7.4. Fetal calf serum (0.2%) was routinely added to the solution.

The experiments were performed at 24°C and 35°C. Bipolar stimuli (0.5 ms duration and 1.5 times threshold strength) were applied by means of two platinum-plate electrodes mounted parallel to the fiber. Fiber bundles were stretched to the length at which tetanic force was maximal corresponding to a mean sarcomere length of 2.57 ± 0.03 µm (n = 17). Resting bundle length, bundle largest and smallest diameters and resting sarcomere length were measured under ordinary light illumination using a microscope fitted with 20× eyepieces and a 5× or 40× dry objective in the experimental chamber and on digital images acquired by a video camera (Infinity Camera, Lumenera Corp., Canada) using image processing software. Mean clip to clip fiber length (*l*_*f*_), including tendons, was 1042 ± 39 µm (n = 17) whereas fiber length (*l*_*0*_) was 710 ± 33 µm (n = 17). Mean length of tendon attachment was therefore 332 µm. Mean cross sectional area, calculated according to the formula π*ab*/4, where *a* and *b* are the smaller and the greater diameters, (both measured at 2-3 different points along the fiber), was 0.083 ± 0.011 mm^2^ (n = 17). Sarcomere length was measured by counting 10 consecutive sarcomeres on a calibrated scale on the acquired images. Stimuli and fiber length changes were controlled by a custom-written software (LabView, National Instruments USA) which was also used to record force and length at sampling speed up to a maximum of 200 kHz. 

Control tetanic contractions of 350 ms duration, 70 Hz stimulation frequency at 24°C and 300 ms, and 100 Hz at 35°C, were elicited every 90 s during equilibration before fatigue, every 1.5 s for 105 consecutive *tetani* during fatigue and every 90 s during recovery from fatigue until force recovered 90-100% of the pre-fatigue value. Fiber force and stiffness at tetanus plateau were measured during the whole experimental protocol. Plateau tetanic force (P_*0*_) during rested contractions was usually stable over the period of the experiment (up to 6 h) but if *P*_*0*_ decreased by >10%, the data were discarded. 

### Analysis of mechanical properties of the preparation and stiffness measurements

To estimate the number of attached crossbridges we measured fiber stiffness [13,14] by applying small sinusoidal length changes (*dl*) at 6.5 kHz frequency to one end of the active fibers and measuring the resulting force oscillations (*dp*) at the other end. Stiffness measurements were expressed relatively to plateau stiffness under rested conditions by the ratio *dp*/*dl* at any tension over *dp*/*dl* measured at *P*_*0*_, or simply by *dp* at any tension over *dp* at *P*_*0*_ since *dl* was strictly constant during each experiment. Mean *dl* peak to peak (p-p) amplitude was 1.88 ± 0.12 µm corresponding to 0.18 ± 0.01% *l*_*f*_ (n = 17). Stiffness measured in this way includes 3 series components: tendon, myofilament and crossbridge stiffness. Therefore it can be directly related to attached crossbridge number only after the correction for the tendon and myofilament contribution. The correction method was explained in details in a previous paper [5] and is explained briefly in the Results section. Stiffness needs to be measured with fast length changes to avoid underestimation due to truncation of the force response by the quick force recovery [15]. At the same time, length changes should not be too fast to produce force artifacts from fiber inertia. The effects of different length oscillation frequency in the range 1-6.5 kHz on stiffness measurements were investigated at 24 and 35°C. The results (not shown here) confirmed those previously published by our group [5] and showed that quick force recovery was negligible at frequencies above 2.5-4 kHz at both temperatures. Even at smaller forces (down to 0.3 *P*_*0*_), phase shift between force and length sinusoids at 6.5 kHz was not different from zero indicating that fiber stiffness measured at this frequency was not significantly affected by quick force recovery or fiber inertia thus representing a reliable measure of the instantaneous elasticity of the fibers. Fiber stiffness was not corrected for passive and static stiffness [16,17] which were considered negligibly small. Force and stiffness data reported in the present paper, were always expressed relatively to the control data before fatigue. 

In the paper, number of crossbridges and individual crossbridge force are used sometimes as synonyms of number of attached crossbridges and average force per attached crossbridge, respectively. 

## Results

### Temperature effects on tension and stiffness

**Figure 1:**
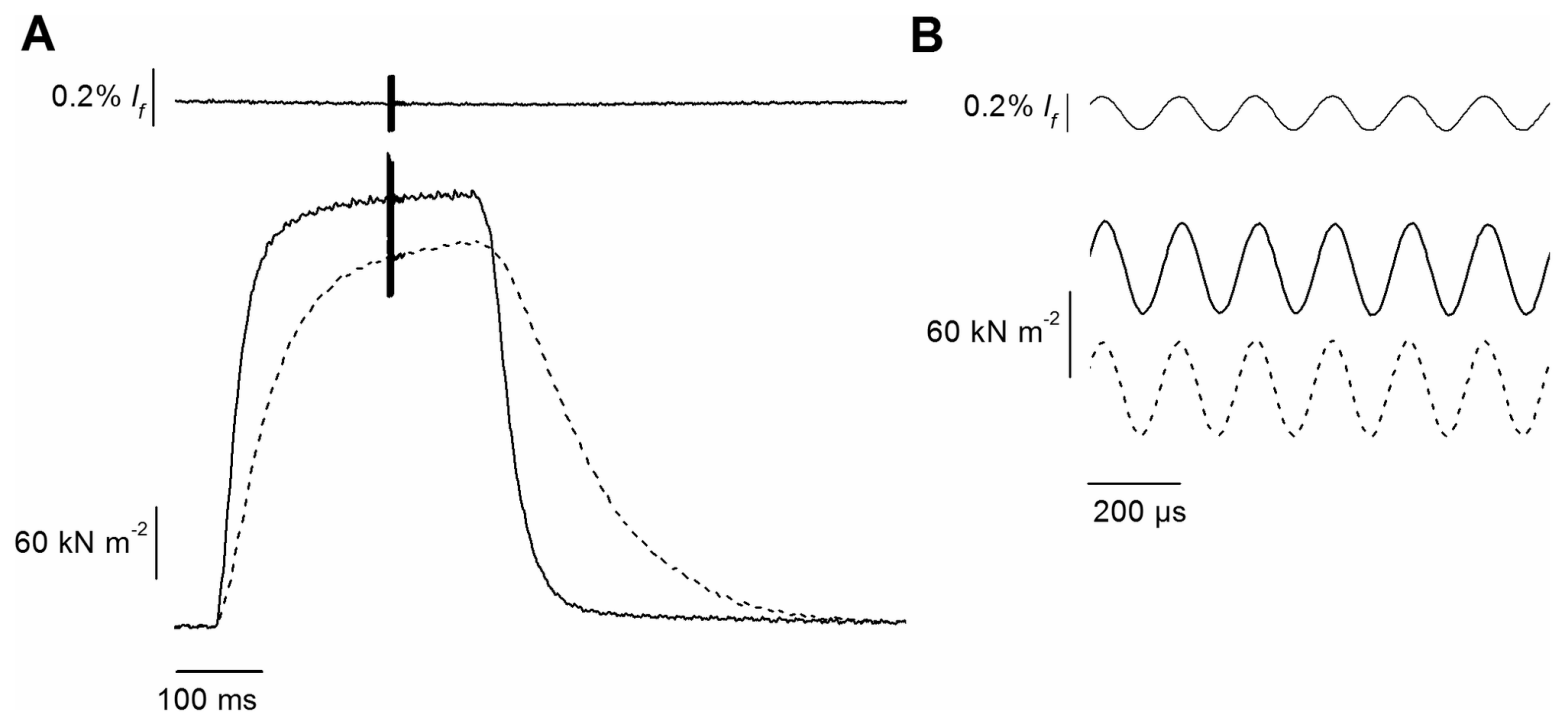
Typical force and length records from an experiments at 24 and 35°C. A, fiber length and superimposed *tetani* at 24 and 35°C (dashed and continuous line, respectively). A portion of the burst of length and force oscillations at 6.5 kHz visible in A is shown at fast time base in B. Note the absence of phase shift between force and length sinusoids. These records were used to measure fiber stiffness. Length oscillation amplitude was 1.90 µm (p-p), corresponding to 0.18% *l*_*f*_.

Both the isometric tension (P_*0*_) and the rate of tetanic tension development increased with temperature as shown in Figure 1. On going from 24 to 35°C, *P*_*0*_ increased by 11 ± 3% (n = 17) from 317 ± 15 kN m^-2^ to 352 ± 22 kN m^-2^ and the half-time for tension development decreased from 41 ± 2 ms to 23 ± 1 ms in agreement with previous data [18].

Measurements on the same group of fibers showed that fiber stiffness at tetanus plateau did not increase significantly from 24 to 35°C, indicating that force potentiation by temperature occurred without changes in attached crossbridge number (proportional to stiffness) consistently with the idea that temperature increases the individual crossbridge force as suggested previously [19,20]. We also measured the ratio (*dl/l*_*f*_)/(*dp*/*P*_*0*_), defined as *y*_*0f*_, which corresponds to the relative extension (% of *l*_*f*_) at the tetanus plateau, of all the elastic components of the fibers: tendon, myofilament and crossbridges. This ratio was used to calculate the contribution of tendon to fiber stiffness (see later). Mean *y*_*0f*_ in 17 fibers, was 0.88 ± 0.05% *l*_*f*_ at 24°C and increased by ~11% to 0.98 ± 0.04% *l*_*f*_ at 35°C. The relative extension of the sole fiber elasticity, excluding tendon, (% of *l*_*0*_) was defined as *y*_*0*_ [15].

### Fatigue

**Figure 2:**
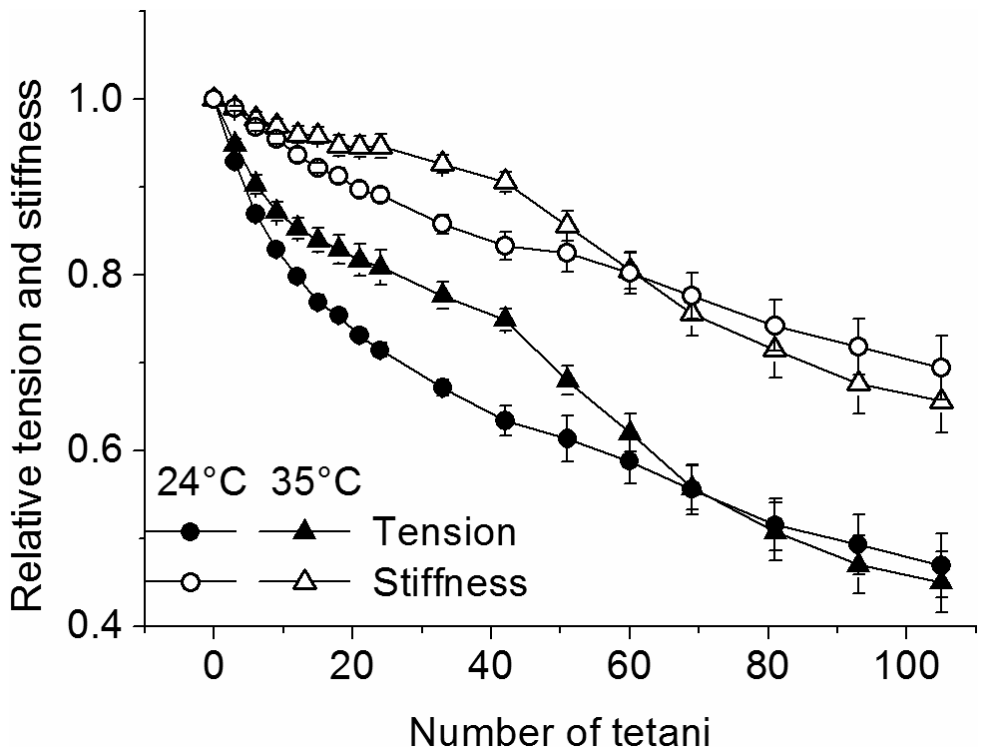
Relative average tension and stiffness changes occurring during fatigue. Tension and stiffness at 24 and 35°C are expressed relatively to plateau control values. Data represent mean ± SEM (n = 9). SEM not visible when smaller than symbols. Note that stiffness falls less than tension at both temperatures.

Fatigue was induced by reducing the time interval between *tetani* from the control value of 90 s to the fatiguing interval of 1.5 s. The decay of relative fiber tension and stiffness during 105 consecutive short *tetani* was measured at 24 and 35°C and it is shown in Figure 2.

**Figure 3:**
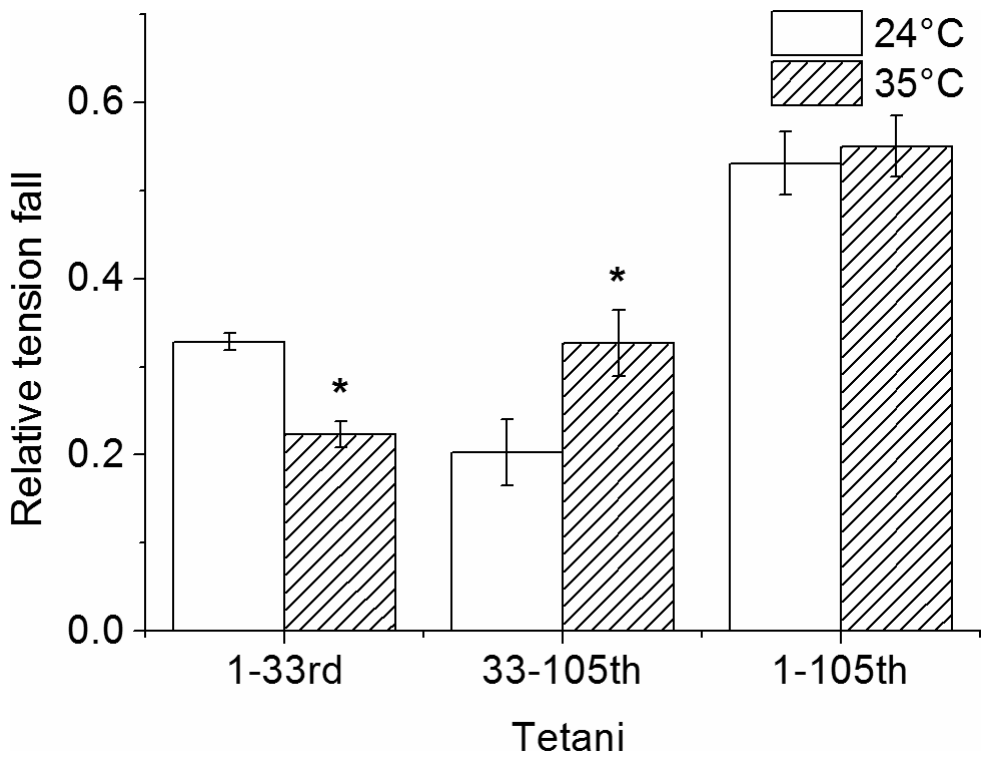
Average tension fall during the two phases of fatigue. Temperature increase reduced the fall of force during initial phase but increased that occurring during later phase. At the end of fatigue the loss of tension was the same at 24 and 35°C. Asterisks indicate statistically significant changes (*P* < 0.05) respect to 24°C.

The decline of both parameters during fatigue is significantly affected by temperature. As shown previously [5], tension and stiffness loss during fatigue can be split into a fast initial phase and a later slower phase. Temperature increase affected in opposite way these two components decreasing the tension decline during phase 1 but increasing that during phase 2. To quantify these observations we compared mean tension changes occurring between the first tetanus and the 33^rd^ and from the 33^rd^ to the 105^th^ at 24 and 35°C and the results are shown in Figure 3. Initial tension loss decreased from 33% to 22% of the control as temperature increased from 24 to 35°C, whereas the decline during phase two increased from 20% to 33%. Because of these opposite effects, the tension at the end of the fatigue was about the same at both temperatures: 0.47 ± 0.04 *P*_*0*_ (n = 9) at 24°C and 0.45 ± 0.03 *P*_*0*_ (n = 9) at 35°C.

Thus, the higher temperature made the fiber more resistant to the early fatigue but increased fatigue during the later phase, consistently with the idea that these phases are determined by two different mechanisms [4-6]. 

### Tetanic tension development

**Figure 4:**
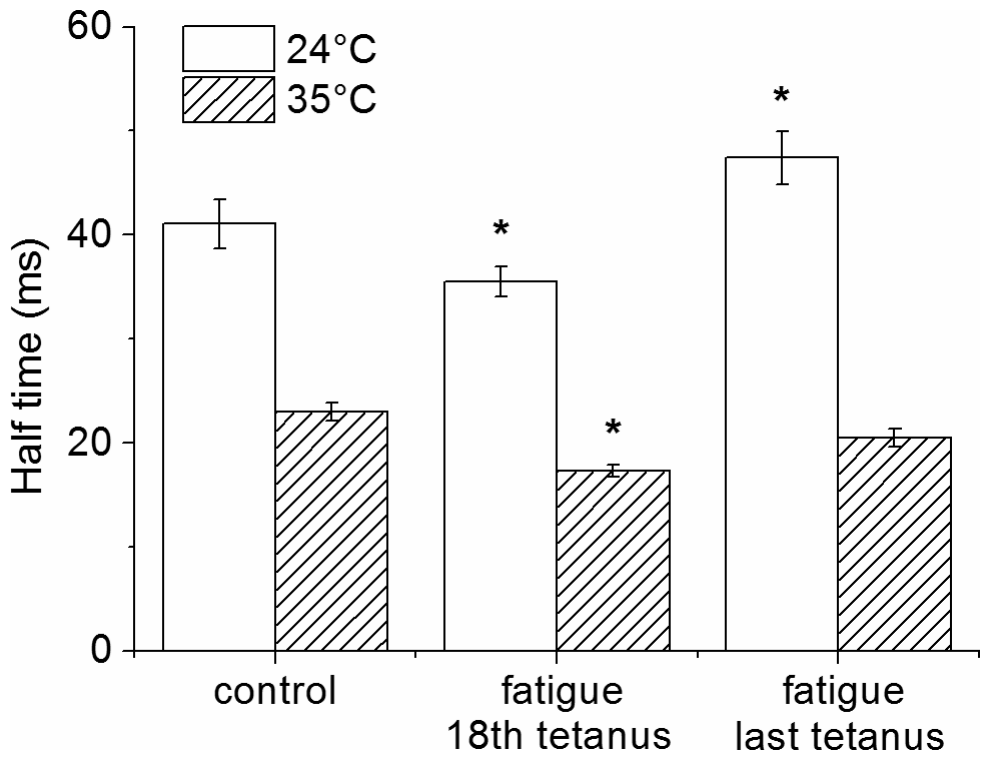
Half-time of tetanic tension rise during fatigue. Measures were made at control tetanus, 18^th^ and 105^th^ tetanus. Asterisks indicate statistically significant changes (*P* < 0.05) respect to control values.

We showed previously that phase 1 was also characterized by a reduction of the half-time of tension development during the tetanus rise [5]. These measures were repeated here at 24°C and extended at 35°C and the results are shown in Figure 4. 

Respect to the control, the half-time of tetanus rise decreased significantly and progressively during phase 1 to reach the minimum value at the 18^th^ tetanus and to rise again up to a value similar to the control at the 105^th^ tetanus. The mean control values at 24 and 35°C were 41 ± 2 ms and 23 ± 1 ms, respectively (n = 9). At the 18^th^ tetanus these values decreased to 35 ± 1 ms and 17 ± 1 ms, respectively, to rise again to 47 ± 3 ms and 20 ± 1 ms at the end of the fatiguing protocols. Thus, the previous finding of a reduction of the half-time of tetanus rise during the initial fatigue is confirmed here and occurs also at 35°C.

### Recovery

**Figure 5:**
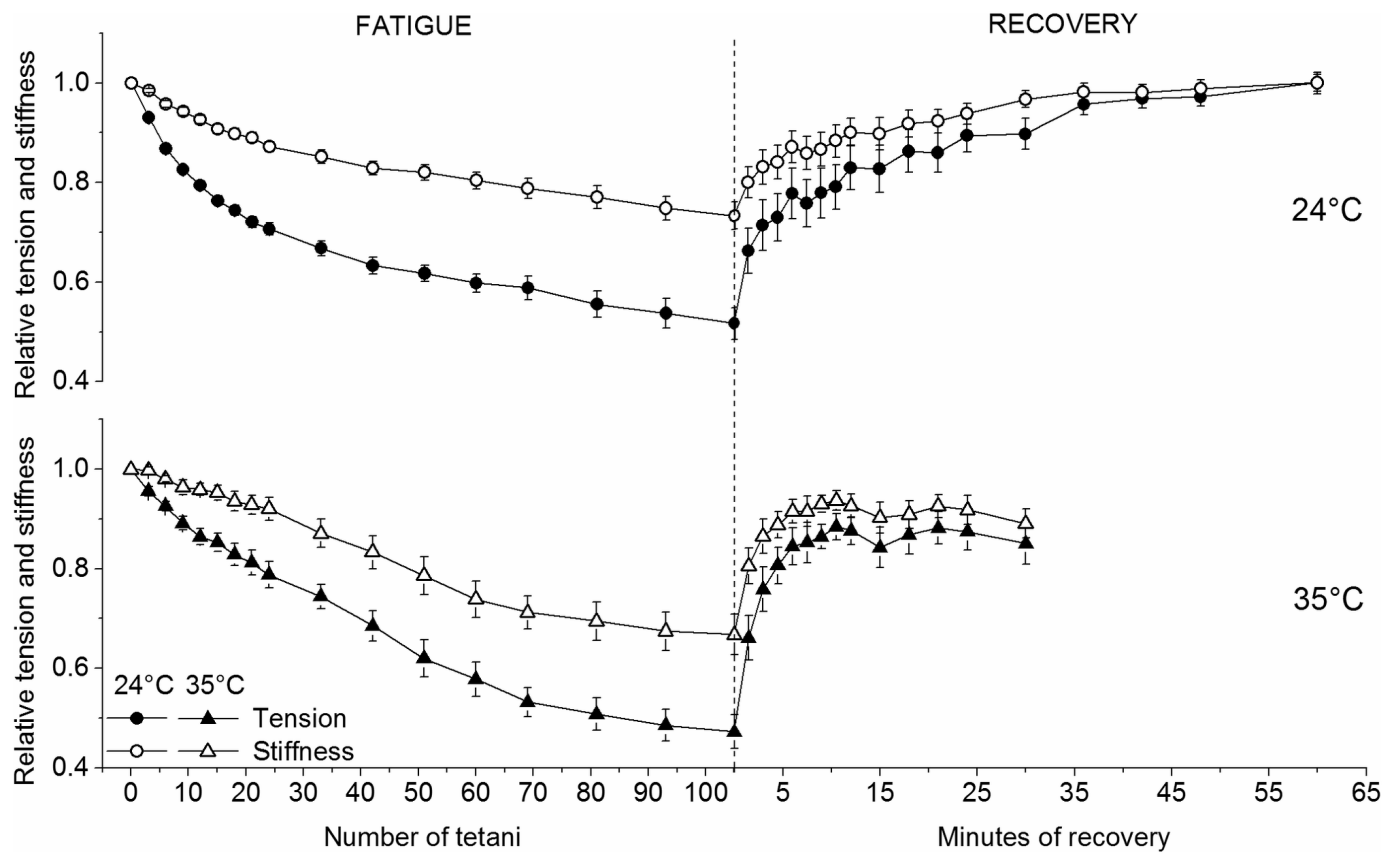
Average time course of tension and stiffness during fatigue and recovery. Experiments were made on a group of bundles (n = 8) different from that used for Figure 2. Empty symbols, stiffness; filled symbols, tension. Note the much faster recovery at 35°C.

After 105 *tetani* of the fatiguing phase, the time interval between repeated *tetani* was switched from 1.5 s back to 90 s thus inducing a period of recovery during which tension and stiffness recovered up to 90-100% of the pre-fatigue value. The average time courses (n = 8) of the changes occurring during recovery at 24 and 35°C and the time course of the corresponding fatigue measured in the same fibers are shown in Figure 5. At 24°C tension and stiffness recovered 100 ± 2% of their pre-fatigue values with an overall half-time of ^~^6 min whereas at 35°C the recovery was 88 ± 3% but it was much faster with an half-time reduced to ^~^1.5 min. Similarly to fatigue, tension recovery could be separated into two phases: a fast initial one followed by a successive slower one. The fast phase lasted few minutes after the end of fatigue whereas the second phase required up to one hour at 24°C and 30 min at 35°C to be complete and showed a relatively great variability. Symmetrically with fatigue, relative stiffness was always greater than relative tension.

### Stiffness and crossbridges properties during fatigue

**Figure 6:**
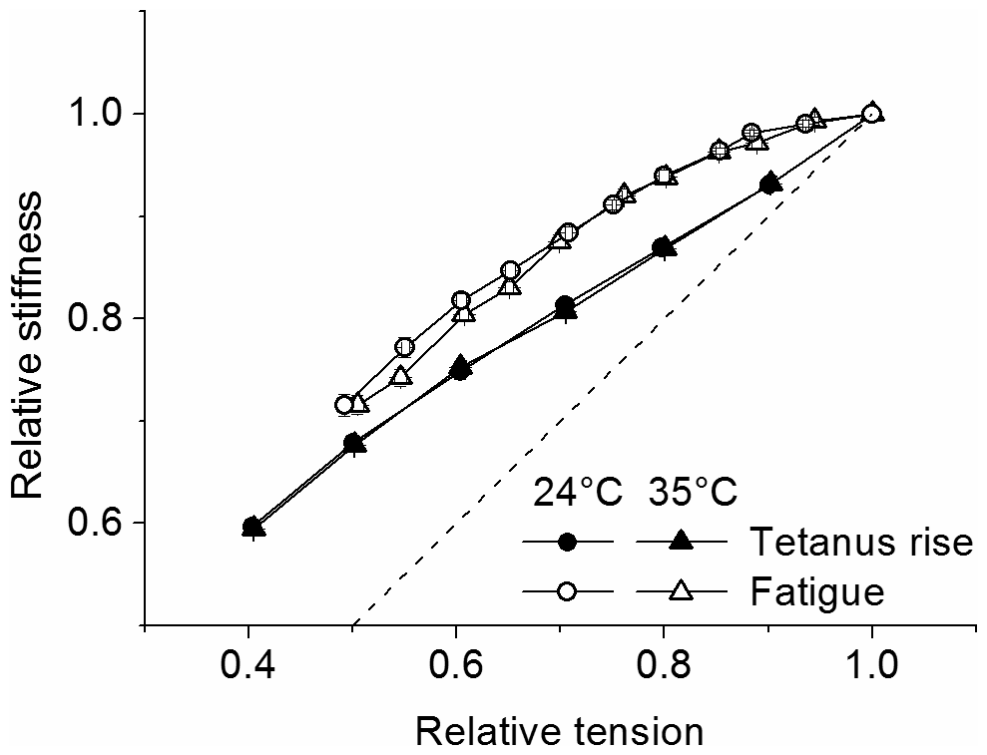
Comparison of the stiffness-tension relations during fatigue and tetanic tension rise. Stiffness and tension are expressed relatively to their plateau values before fatigue. Data during fatigue are the same of Figure 2. The dashed straight line indicates the direct proportionality between tension and stiffness. Measurements on the tetanus rise (filled symbols) and fatigue (empty symbols) were made on the same fiber.

The loss of muscle force during fatigue can be due to a reduction of attached crossbridge number and/or a decrease of the individual crossbridge force. Changes in attached crossbridge number are proportional to crossbridge stiffness and they can be obtained from fiber stiffness measurements after the correction for the contribution of tendon and myofilament stiffness [21,22]. The effects of tendon and myofilament stiffness can be shown by plotting the fiber stiffness-tension relation (S/T relation) obtained from measurements taken during the tetanus rise in rested fibers (Figure 6). During the tetanus rise, in fact, crossbridge stiffness is directly proportional to tension [23] hence, if fiber stiffness were due exclusively to the crossbridges, we would expect the S/T relation to be linear with a unitary slope. Figure 6 (filled symbols) shows instead that the relation is not linear and deviates from the direct proportionality (showed by the dashed line) as relative stiffness is always greater than relative tension [23]. These effects are precisely the result of the myofilament and tendon stiffness presence. Clearly, tendon and myofilament effects are also present during fatigue and they need to be corrected to find out crossbridge stiffness alone.

**Figure 7:**
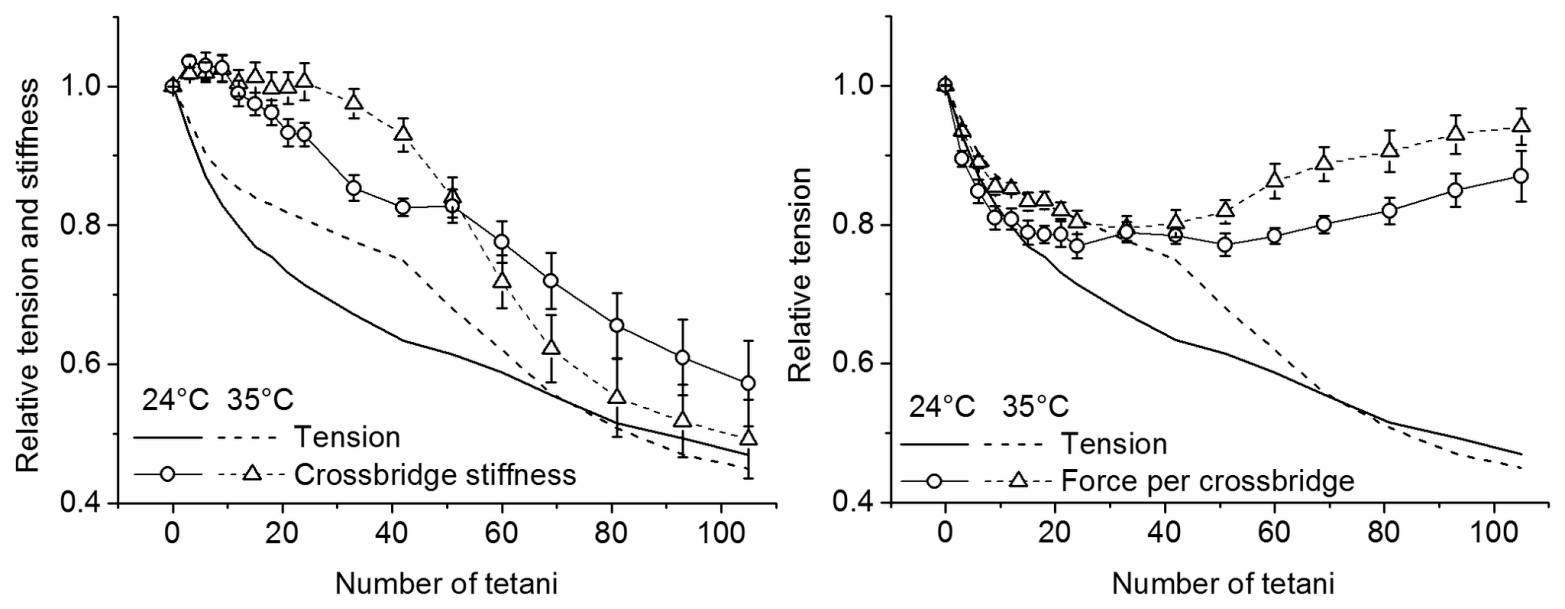
Tension, crossbridge stiffness and average force per crossbridge during fatigue. Note (left) that the period during which crossbridge stiffness remains almost constant in spite of the fall in tension (continuous and dashed lines, same data of Figure 2), is much longer at 35°C (triangles) than at 24°C (circles). During this phase (right) at both temperatures almost all the drop of force continuous and dashed lines) is accounted for by a reduction of the average force per crossbridge (open symbols).

Qualitatively, we can observe that if tension loss during fatigue were due exclusively to reduction of attached crossbridge number, the S/T relation during fatigue should be exactly the same as during the tetanus rise. The data of Figure 6 show that this is not the case: during fatigue, in fact, the S/T relation (empty symbols) has a more pronounced downward curvature and deviates even more from linearity so that, at any tension, fiber stiffness is greater than during the tetanus rise. Since filament and tendon stiffness are the same in both conditions this means that at any given force, to justify the greater fiber stiffness, there must be more attached crossbridges during fatigue than during the tetanus rise, which means that each fatigued crossbridge generates less average force than controls. This effect is greater during the initial phase of tension loss and tends to decrease at lower tension and it is very similar at 24 and 35°C. To quantify these observations, we calculated the crossbridge stiffness alone during fatigue with a method described in details in a previous work [5] using the stiffness-tension data of Figure 6. Briefly, crossbridge compliance (the reciprocal of crossbridge stiffness) was calculated by subtracting tendon and myofilament compliances from the fiber compliance. Tendon compliance could not be measured directly as done previously [24,25] and its relative contribution to rested fiber compliance at tetanus plateau, was calculated by the (*y*_*0f*_ *l*_*f*_ - *y*_*0*_ *l*_*0*_) /*y*_*0f*_ *l*_*f*_ ratio. *Y*_*0f*_, which represents the extension of the fiber compliance with tendon, was measured as described, whereas *y*_*0*_, representing the extension of fiber compliance without tendons (filament plus crossbridge compliance), was extrapolated from data in literature [26]. Successively, by knowing (i) tendon compliance, (ii) that the relative contribution of myofilament and crossbridge compliance to fiber compliance is 38% and 62% respectively [26] and (iii) that crossbridge compliance during the tetanus rise is inversely related to tension [23], we calculated crossbridge and filament compliances of our fibers. The results were the same at 24 and 35°C and showed that at tetanus plateau, tendon, filament and crossbridges accounted for ^~^41%, ^~^22% and ^~^37% respectively, of fiber compliance. Finally, tendon and filament compliances were subtracted from fiber compliance measured during fatigue to obtain the crossbridge compliance and its reciprocal crossbridge stiffness. Average force per crossbridge was then given by the ratio relative tension/crossbridge stiffness. The results of these calculations are shown in Figure 7.

It can be seen (Figure 7, left) that during the early phase of fatigue, force falls without changes of crossbridge stiffness (or attached crossbridge number) indicating a reduction of individual crossbridge force, in agreement with previous results [5]. This period lasted longer at 35°C than at 24°C ending after ~24 and ~12 *tetani*, respectively. During the later phase, stiffness decreased in proportion to tension indicating a reduction of crossbridge number. The greater fall occurring at 35°C suggests a greater reduction of the number attached crossbridges at high temperature during this phase. Figure 7 (right) shows the time course of the calculated average force per crossbridge at the two temperatures. During the early phase of fatigue, the force per crossbridge decreased less at 35 than at 24°C. At the 15^th^ tetanus at 24°C, for example, relative crossbridge force was down to 0.79 ± 0.02 (n = 9) whereas at 35°C force was down to 0.83 ± 0.01 (n = 9). This change, statistically significant (p<0.05), means that force decline during phase 1 was ^~^19% smaller at 35 than at 24°C, suggesting a protective role of the high temperature on crossbridge individual force. The time course of the average force per crossbridge (Figure 7, right) shows that the minimum force is reached after 20-25 *tetani*, significantly earlier than the 33^rd^ tetanus taken as the end of phase 1 in Figure 3. This is because the initial fiber force loss, especially after 20 *tetani*, is influenced also by the reduction of attached crossbridge number with a consequent effect on the force time course. It is interesting that maximum reduction of the half-time of tetanus rise due to the same mechanism which induces the crossbridge force reduction (see discussion), occurs after the 18^th^ tetanus, almost at the same time of the minimum average force per crossbridge. The crossbridge force lost during phase 1 is partially recovered during phase 2 and the recovery is greater at 35°C than at 24°C, maintaining the force per crossbridge always higher at 35°C than at 24°C.

### Stiffness and crossbridge properties during force recovery

**Figure 8:**
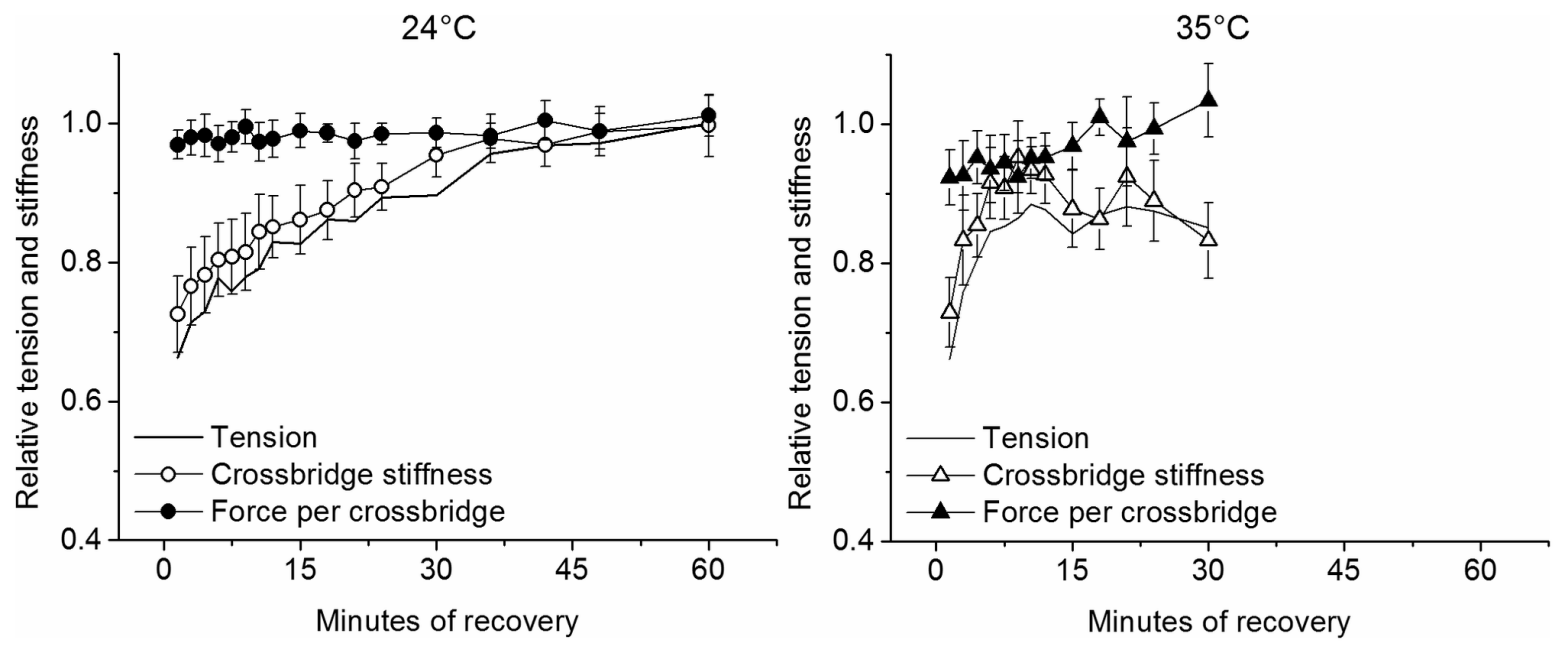
Time course of average force per crossbridge, crossbridge stiffness and tension during recovery at 24 and 35°C. Note that the recovery of the average force per crossbridge is already complete 90 s after the end of fatigue at both temperatures whereas the recovery of attached crossbridge number requires a much longer time.

The analysis above was applied also to force recovery after fatigue at 24°C and 35°C and the results are shown in Figure 8. It can be seen that at both temperatures the average force per attached crossbridge is completely recovered at the time of the first test tetanus evoked 90 s after the end of fatigue. The successive later force recovery is much faster at 35 than 24°C and it is accompanied by a parallel increase of crossbridge stiffness, indicating a recovery of the pre-fatigue crossbridge number. Thus, the two phases of the recovery likely correspond to the reversal of phase 1 and 2 of fatigue. 

## Discussion

The main purpose of the present study was: (i) to test whether crossbridge properties modifications occurring during fatigue in intact FDB fiber bundles are affected by temperature in the range 24-35°C; (ii) to test the characteristics of force recovery following fatigue at 24 and 35°C. Our major findings are: (i) temperature increase from 24 to 35°C affected phase 1 and 2 of fatigue in opposite way: prolonged and attenuated the tension decline during phase 1 but increased the tension decline during phase 2; (ii) force recovery at both 24 and 35°C could be split into two phases symmetrical to those observed during fatigue. Phase 1 was complete in few minutes after the end of fatigue and was due to the recovery of the individual crossbridge force; the second phase was much longer and corresponded to the recovery of the pre-fatigue attached crossbridges number. The recovery of tension during phase 2 was much faster at 35 than at 24°C. We also found that temperature increase from 24 to 35°C did not affect fiber stiffness, indicating that tetanic force potentiation by temperature, in this range, is mainly due to the increase of the average force per crossbridge in agreement with our previous findings [19,20].

### Fatigue at 24 and 35°C

The increase of temperature from 24 to 35°C, a temperature occurring in mouse FDB muscle during a prolonged physical exercise [7-9], did not induce any premature effect on fatigue. Actually, force during phase 1 decreased less at 35°C indicating that temperature has a beneficial effect on this phase of fatigue, in agreement with a previous report on rat muscle [27]. In addition, at 35°C, the instauration of phase 2, during which force fall increased, was delayed so that phase 1 was prolonged. In contrast, temperature accelerated and increased the force decline during phase two. These two opposite effects compensated each other so that the overall force decline at the end of fatiguing protocol of 105 *tetani* was about the same at 24 and 35°C. It is interesting that two opposite effects on force decline during fatigue were also produced by the application of high hydrostatic pressure on frog muscle fibers. The high pressure depressed the tetanic tension during the early phase of fatigue but potentiated the tension at later stages [28]. The finding that tension loss at the end of fatigue was the same at 24 and 35°C is in agreement with previous studies, which did not consider the different phases of fatigue, reporting little or no effect of temperature on fatigue [9,27,29].

### Mechanism of force decline during fatigue

Figure 2 shows that force fall during phase 1 at both 24 and 35°C is accompanied by a much smaller decrease of fiber stiffness. Calculations showed that crossbridge stiffness (proportional to the number of attached crossbridges) remained almost constant (see Figure 7) during this period indicating that force fall was due mainly to a reductions of the average force per attached crossbridge in agreement with previous results at 24°C [5]. Force per crossbridge decreased less at 35 than at 24°C for the whole fatiguing protocol: with respect to control, force at the 15^th^ tetanus was down by ^~^21% at 24°C, and by ^~^17% at 35°C. These values are subjected to some uncertainties due to the assumptions made in our calculation, however these uncertainties do not alter the conclusions that the fall in force during early fatigue is due to a reduction of the average force per attached crossbridge and that this reduction is smaller at higher temperature, suggesting again a protective role of temperature on the average crossbridge force fall during phase 1. 

Tetanic [Ca^2+^]_i_ did not decrease during the initial *tetani* but it increased slightly [30,31]. Thus, intracellular Ca^2+^ decrease does not seem the reason underlying the early loss of average force per crossbridge. A change of intracellular pH is also unlikely since during the initial part of fatigue pH showed little change [8,32]. Previous studies using NMR have demonstrated that during a bout of contractile activity comparable to that used in this study, there is little change in [ATP] while both [P_i_] and [ADP] more than double [33-35]. Increased [ADP] appears to play little role in modulating crossbridge function [36,37], however it is clear that increased [P_i_]_i_ has a depressant effect on force development [1,37] The depressant effect of P_i_ decreases with temperature and it is relatively small near physiological temperature [1-3]. The values reported for 25-30 mM of internal [P_i_], a concentration reached during a prolonged exercise, varied between 5% and 30% *P*_*0*_. These values can be compared with our mean value of 22% P_0_ of tension loss in intact preparations during phase 1 of fatigue at 35°C (Figure 3). The precise mechanism by which P_i_ inhibits force production is still not completely understood. A simple kinetic scheme suggests that elevated [P_i_]_i_ increases the back reaction for P_i_ release pushing the crossbridges into a low or no force-generating state [36,38,39]. This mechanism is expected to reduce the average force per attached crossbridge without affecting the attached crossbridge number or stiffness and it is therefore consistent with our findings during phase 1. Further important indications in favor of the P_i_ hypothesis are: (i) the reduction of the half-time of the tetanus rise found experimentally (this paper, and [5]) and (ii) the reduction of fatigue during phase 1 induced by high temperature. The first effect is expected on kinetic bases if the back reaction postulated above really occurs at high [P_i_]_i_ whereas the second effect is expected from experiments on skinned fibers showing that P_i_-induced force depression is mitigated at high temperature [2]. It is important to note that this last effect occurred in spite of the higher P_i_ production due to the increased ATPase rate at 35°C compared to 24°C. In our experiments this effect was not compensated by an adequate reduction of the tetanus duration [27]. Finally, kinetics of force fall and recovery of phase 1 are consistent with ^31^P-MRS studies showing that [P_i_]_i_ increase during exercise reaches a steady level in ^~^90 s and falls at the end of the exercise with a similar time course [40].

The mechanism of force decline during phase 2 differs from that of phase 1. This conclusion follows from the observations that tension decline during phase two (i) is accompanied by a parallel decline of crossbridge stiffness and (ii) it is greater at high temperature, in contrast with the findings during phase 1. The loss of crossbridge stiffness in parallel with force suggests that the mechanism of fatigue implies mainly a reduction of attached crossbridge number. Two mechanisms acting synergically could be involved in this reduction: the first one is the impairment of calcium release from sarcoplasmic reticulum caused by the exposure of myofibrils to high [P_i_]_i_. This effect could be due to calcium phosphate precipitation inside the sarcoplasmic reticulum which occurs in skinned fibers even after a short exposure time (2 min) to the P_i_ concentration reached during fatigue protocol (30 mM) [41]. The second mechanism is the inhibitory effect of high [P_i_]_i_ on myofibrillar calcium sensitivity. It has been shown, in fact, that P_i_ at high concentrations, shift to the right the pCa-tension curve in skinned fibers reducing the calcium sensitivity of the myofibrils [2]. This effect is greater at high temperature and it overcomes the increased myofibrillar sensitivity to Ca^2+^ induced by high temperatures [2]. In combination with the decrease of tetanic [Ca^2+^]_i_, described above, this effect could decrease substantially muscle force output [6]. A reduction in calcium sensitivity or calcium release reduces the activation of the fibers and it is expected to reduce attached crossbridge number (or stiffness) and thus this mechanism would be consistent with our finding during phase 2. However, the decrease of Ca^2+^ release or sensitivity needs to have slow kinetics similar to our phase two which requires several minutes to reach the equilibrium. This characteristics has been observed in many isolated muscle protocols [31]. The mechanism by which increased P_i_ concentration reduces Ca^2+^ sensitivity is not clear. It has been proposed that elevated [P_i_]_i_ either inhibits Ca^2+^ binding to TnC [42] or antagonizes Ca^2+^ action enhancing the strong to weak crossbridge state transition [43]. However, both these effects seem too fast compared to the relatively slow time course of phase two. Another possibility regards the effects of ROS/RNS produced during fatigue on muscle force generation. Results in literature are somewhat contrasting, showing either a role or no detectable effects of ROS on force production [44]. When present, the effects of ROS/RNS were more prominent at submaximal than near-maximal forces, indicating that they mainly involve changes in muscle activation, very likely a reduction of myofibrillar Ca^2+^ sensitivity [44]. Thus, it is possible that ROS are responsible, at least partially, for the force loss during phase two of fatigue. The increase of force loss during phase two at high temperature would be consistent with the higher production of ROS/RNS.

### Mechanism of force recovery after fatigue

As fatigue, force recovery could be split in two part: the early and late recovery. Similarly to fatigue the two components have a different kinetics and are attributable to different mechanisms. The early tension recovery at both 24 and 35°C was characterized by the recovery of the average force per attached crossbridge suggesting that it represents the reversal of the crossbridge force loss occurring during early fatigue. Thus, this phase of recovery is likely due to the return of the internal P_i_ concentration, increased during fatigue, to the pre-fatigue value. This hypothesis is consistent with the observation that the kinetics of the return of internal [P_i_] to pre-fatigue concentration shown by ^31^P-MRS measurements after exercise in human muscle [40], is similar to that of phase 1 force recovery and with many observations in literature [4]. The phase two of recovery was accompanied by a parallel crossbridge stiffness increase, symmetrical to the crossbridge stiffness loss during phase two of fatigue. This is likely associated to the return of Ca^2+^ sensitivity and/or Ca^2+^ release to the normal level following [P_i_]_i_ return to pre-fatigue value. The recovery mechanism is much faster at high temperature with an overall half-time of ^~^1.5 min at 35°C compared to an half-time of ^~^6 min at 24°C. Phase 1 of recovery is already complete by 1.5 min after the end of fatigue, at the time of application of the first test tetanus. The remaining time is taken by phase 2 as shown in Figure 8. It is important to point out that the entire phase two of force recovery, which may require between 10 min and 1 hour to be complete (depending on the temperature and on the fiber), occurs when P_i_ concentration had already recovered the resting value, as shown by the full recovery of the average force per crossbridge force and ^31^P-MRS data [40]. This indicates that the recovery of Ca^2+^ release and/or myofibrillar Ca^2+^ sensitivity during the phase 2 of recovery needs to occur with a relatively long delay compared to [P_i_]_i_ to explain the time course of the recovery. Thus it is possible that P_i_ concentration acts on Ca^2+^ release or sensitivity with an indirect mechanism characterized by a relatively slow kinetics. It is also possible that the recovery is affected by the return of ROS/RNS concentration to resting values after fatigue. The effects of ROS/RNS on muscle force are mostly exerted through a reduction of Ca^2+^ myofibrillar sensitivity and therefore they would be in agreement with our finding on phase 2 of recovery. Whatever the mechanism, it needs to be very sensitive to muscle temperature as shown by the much faster tension recovery at 35°C compared to 24°C, which indicates a Q10 of ^~^3.

At 35°C the force recovery was not complete leaving a force deficit of ^~^10% which is attributable to phase 2 since force fall during phase 1 of fatigue was smaller at high temperature. The force deficit as well as the recovery time course at 35°C are consistent with previous data at 37°C [29]. The reason of the incomplete recovery is unclear. It can be observed that 35°C is a physiological temperature for FDB muscle during continued exercise *in vivo*, but it is higher than the *in vivo* temperature during recovery phase. This might induce a failure of complete recovery, perhaps due to the higher ROS/RNS production which has been shown to reduce force after a prolonged exposure [45].

In conclusion, the results of our experiments on intact muscle bundles indicated that the low intensity reversible fatigue is sensitive to temperature. However, given the opposite effects of high temperature on the early and the later phase of fatigue, the increase of temperature from 24 to 35°C has almost no effect on the final force reached at the end of a fatiguing protocol of 105 repeated short *tetani*. The beneficial effect of high temperature on the early phase of fatigue, which manifest itself as a smaller loss of the average force per attached crossbridge, is attributable mainly to the attenuated inhibitory effect of the increased [P_i_]_i_ on force at high temperature. Whereas the increased fatigue occurring during phase 2 is likely due to the combined action of reduction of Ca^2+^ sensitivity and Ca^2+^ release due to increased [P_i_]_i_ and/or ROS/RNS production at high temperatures. Other effects, due for example to changes of pH, ADP etc. are considered unlikely, although they cannot be completely ruled out. This study agrees with data in literature indicating that high temperature does not accelerate fatigue development in fast twitch fiber [9] and confirms that reduction in performance during exercises *in vivo* at high temperature is not due to intrinsic factors of the muscle fibers [46-50].
